# The Risk Factors of Blood Cadmium Elevation in Chronic Kidney Disease

**DOI:** 10.3390/ijerph182312337

**Published:** 2021-11-24

**Authors:** Kai-Fan Tsai, Pai-Chin Hsu, Chia-Te Kung, Chien-Te Lee, Huey-Ling You, Wan-Ting Huang, Shau-Hsuan Li, Fu-Jen Cheng, Chin-Chou Wang, Wen-Chin Lee

**Affiliations:** 1 Division of Nephrology, Department of Internal Medicine, Kaohsiung Chang Gung Memorial Hospital and Chang Gung University College of Medicine, Kaohsiung 83301, Taiwan; b9302095@cgmh.org.tw (K.-F.T.); nick9335@cgmh.org.tw (P.-C.H.); ctlee33@cgmh.org.tw (C.-T.L.); 2 Department of Emergency Medicine, Kaohsiung Chang Gung Memorial Hospital and Chang Gung University College of Medicine, Kaohsiung 83301, Taiwan; g00308@cgmh.org.tw (C.-T.K.); a0953283092@cgmh.org.tw (F.-J.C.); 3 Department of Laboratory Medicine, Kaohsiung Chang Gung Memorial Hospital and Chang Gung University College of Medicine, Kaohsiung 83301, Taiwan; youhling@cgmh.org.tw (H.-L.Y.); huangminnie@cgmh.org.tw (W.-T.H.); 4 Division of Hematology-Oncology, Department of Internal Medicine, Kaohsiung Chang Gung Memorial Hospital and Chang Gung University College of Medicine, Kaohsiung 83301, Taiwan; lee0624@cgmh.org.tw; 5 Department of Occupational Medicine, Kaohsiung Chang Gung Memorial Hospital and Chang Gung University College of Medicine, Kaohsiung 83301, Taiwan; ccwang52@cgmh.org.tw

**Keywords:** cadmium, chronic kidney disease, risk factors

## Abstract

Low-level cadmium exposure has adverse effects on chronic kidney disease (CKD); however, the risk factors for elevated blood cadmium levels (BCLs) have not been studied in CKD. We conducted a cross-sectional investigation in 200 CKD patients and stratified them by the tertiles of BCL to compare their demographic, environmental, and biochemical data. The factors associated with BCL were identified, and their effects were examined in subgroups. In the analyses, female sex, smoking, and CKD stage 5D were associated with high BCL, and statin was inversely correlated with BCL (odds ratio [95% confidence interval, CI], 6.858 [2.381–19.746], *p* < 0.001, 11.719 [2.843–48.296], *p* = 0.001, 30.333 [2.252–408.520], *p* = 0.010, and 0.326 [0.122–0.873], *p* = 0.026; deviations of BCL [nmol/L, 95% CI], 2.66 [1.33–4.00], *p* < 0.001, 3.68 [1.81–5.56], *p* < 0.001, 3.38 [0.95–5.82], *p* = 0.007, and −2.07 [−3.35–−0.78], *p* = 0.002). These factors were also independently correlated with BCL in subgroups, including non-dialysis CKD, hypertensive patients, non-smokers, and male patients. In conclusion, female sex, smoking, and CKD stage 5D were the major risk factors for elevated BCL; additionally, statins were negatively associated with BCL in CKD.

## 1. Introduction

Cadmium, a toxic heavy metal derived from agricultural and industrial sources, has multiple adverse effects on human health, including chronic kidney disease (CKD), bone disease, cardiovascular disease, infertility, and malignancy. With a long half-life of up to 30 years, cadmium accumulates in the kidney, liver, and bone, and the impact on the health could be long-lasting [1,2]. In addition to high-level exposure via occupational sources or pollution events [3,4], there has been growing evidence supporting the association between low-level environmental cadmium exposure and diseases, such as dyslipidemia, hypertension, lung disease, and renal disease [5,6,7,8]. The possible environmental sources of cadmium include contaminated food or water, cigarette smoking, unqualified cosmetic products or paint, emissions from mining or smelting, phosphate fertilizers, fuel combustion, disposal of metal wastes, and relevant manufacturing processes [9,10,11]. Other risk factors associated with high body cadmium storage in the general population have also been identified in different studies, such as old age, smoking, dietary preference, female sex, and iron deficiency [12,13]. In comparison with the Western population, cadmium exposure seems more common in individuals from South Asia and East Asia according to epidemiologic studies [12]. In Taiwan, the cadmium pollution of farmlands related to industrial sources has been a public concern, and previous research indicated that the overall cadmium exposure might be more prominent in Taiwan than in America or Germany [14]. On the other hand, CKD is a prevalent disease worldwide, affecting 11–13% of the population, and a link between low-level environmental cadmium exposure and the risk of CKD has been found in the literature [15,16,17]. In Taiwan, the prevalence of CKD approaches 15%, and prevalent cases of end-stage renal disease (ESRD) reached 3587 per million population in 2018, commonly recognized as the most prevalent in the world [18,19]. In addition, previous reports indicated that blood and urinary cadmium levels were positively correlated with proteinuria and the risk of CKD in Taiwan [20,21]. Furthermore, the association between the blood cadmium concentrations and risk of mortality in ESRD patients, including hemodialysis (HD) and peritoneal dialysis (PD), has also been observed [22,23]. Altogether, low-level cadmium exposure from environmental sources is relatively common and has adverse impacts on both the risks and outcomes of CKD, and the avoidance of cadmium exposure should be emphasized in the CKD population. However, the risk factors associated with cadmium exposure and elevated blood cadmium concentrations have yet to be specifically studied in CKD patients, and the relationship between blood cadmium concentrations and CKD severity has not yet been discussed.

In this cross-sectional investigation, we assessed the blood cadmium in CKD patients and analyzed the demographic, environmental, lifestyle, and biochemical risk factors of elevated blood cadmium concentrations in the CKD population.

## 2. Materials and Methods

### 2.1. Patient Enrollment

The study protocol was approved by the Institutional Review Board and Ethics Committee of Chang Gung Medical Foundation, Taipei, Taiwan (IRB No. 202001027B0) and adhered to the principles of the Declaration of Helsinki and Declaration of Istanbul. All the patients provided informed consent. The patients living in geographically different areas in southern Taiwan were recruited from the CKD health consultation unit and the HD center in Kaohsiung Chang Gung Memorial Hospital between December 2020 and March 2021. The inclusion criteria were as follows: (1) adult patients (≥20-year-old) with CKD stage 3a–5D (5D indicating those with CKD stage 5 requiring maintenance dialysis) and (2) patients receiving follow-up treatment for at least one year in the nephrology outpatient department or HD center of the hospital. Patients with a history of occupational or accidental heavy metal poisoning, liver cirrhosis, malignancy under chemotherapy or radiotherapy, active infectious disease, active alcoholism, PD, or drug abuse were excluded. Pregnant patients and those who were hospitalized within 3 months of enrollment were also excluded.

### 2.2. Demographic, Environmental and Lifestyle Profile Collection

The demographic data of the eligible patients were collected at the enrollment visit and from the electronic medical record system of the hospital, including age, sex, body mass index (BMI), CKD staging, comorbidities, such as hypertension, diabetes mellitus, dyslipidemia, vascular disease, heart failure, chronic obstructive pulmonary disease (COPD), gout or hyperuricemia, degenerative or osteoporotic bone disease, history of malignancy, and transplantation history. CKD staging was defined according to the Kidney Disease Improving Global Outcomes 2012 Clinical Practice Guideline for the Evaluation and Management of CKD [24] and based on two consecutive serum creatinine (SCr)-based estimated glomerular filtration rate (eGFR) data (one at the enrollment visit and another within 3 months prior to enrollment). The Modification of Diet in Renal Disease equation, which is eGFR (mL/min/1.73 m^2^) = 175 × SCr^−1.154^ × age^−0.203^ × 0.742 (if female), was used to retrieve the eGFR [25]. Hypertension was defined as the regular use of at least one antihypertensive agent or at least two blood pressure measurements above 140/90 mmHg. Diabetes was defined as the regular use of at least one glucose-lowering agent or at least two consecutive tests of glycated hemoglobin (HbA1c) level > 6.5%. Dyslipidemia was defined as the regular use of a lipid-lowering agent or at least 2 consecutive tests of abnormal lipid profiles (i.e., total cholesterol ≥ 5.18 mmol/L, low-density lipoprotein cholesterol ≥ 3.37 mmol/L, high-density lipoprotein cholesterol ≤ 1.04 mmol/L, or triglyceride ≥ 1.69 mmol/L). Vascular diseases, including cardiovascular, cerebrovascular, carotid, and peripheral vascular diseases, and other comorbidities, were extracted from the medical records. Long-term medications continuously prescribed for at least 3 months, including antihypertensives, glucose-lowering agents (oral or injection), and statins, were also recorded. A questionnaire (Supplementary Table S1) was provided to collect the environmental and lifestyle profile of each patient at the enrollment visit, such as smoking, drinking, betelnut and herb usage, dietary preference, daily traffic style, current and previous occupations, address and house age of residence, drinking water source for daily life, and usage of cosmetics. Since most people in Taiwan consume rice or rice products as the staple food, the dietary survey focused on food consumption related to heavy metal exposure reported in studies, such as seafood and organ meat [12]. Considering the long half-life of cadmium, drinking, smoking, betelnut, herb, and cosmetics usage were recorded according to the exposure history within 10 years prior to enrollment. In southern Taiwan, Kaohsiung City is a major municipality and includes both urban and rural areas, and there are several petrochemical industrial regions distributed in the city. The address of residence was utilized to determine whether the patient lived in an urban and petrochemical industrial region. The traffic style, occupation, house age of residence, and source of drinking water were all collected to evaluate each patient’s proximity to relevant daily sources of heavy metals, such as air pollution, occupational contact, peeling house painting, and contaminated water. For CKD stage 5D patients, reverse osmosis (RO) water samples from the HD center were collected and examined for cadmium levels in 2020 and 2021 to exclude HD-related cadmium exposure, according to the routine schedule of the HD center, which was performed by the laboratory of Tze-Chiang Foundation of Science and Technology (Hsinchu City, Taiwan), which is qualified for dialysis water analysis in Taiwan.

### 2.3. Blood and Urinary Biochemical Profile Measurement

The blood biochemical data including SCr, eGFR, hemoglobin, HbA1c, lipid profiles, liver enzymes, serum electrolytes, uric acid, serum albumin, and the transferrin saturation were measured at the enrollment visit. For non-dialysis CKD patients, the first-void urine in the morning was collected within one week after the enrollment visit for urinary protein/creatinine ratio (UPCR).

### 2.4. Inductively Coupled Plasma Mass Spectrometry (ICP-MS) for Blood Cadmium

The blood samples for cadmium measurements were collected during enrollment. The specimens were collected in 3 mL plastic collection tubes containing dipotassium ethylene diamine tetraacetic acid (K_2_EDTA) anticoagulant (BD, Franklin Lakes, NJ, USA) and stored at 4 °C. The blood cadmium level (BCL) was quantified using ICP-MS on an Agilent 7800 ICP-MS instrument (Santa Clara, CA, USA) and analyzed using a no-gas mode. Blood specimens (500 μL) were diluted (1 + 9) with a 1.5% nitric acid (JT Baker, Phillipsburg, NJ, USA) solution containing yttrium as an internal standard. The cadmium and yttrium standards were purchased from AccuStandard (New Haven, CT, USA; standard range = 2.67–355.84 nmol/L). The calibration curve had an R ≥ 0.995. The BIO-RAD Lyphochek^®^ Whole Blood Metal Control Levels 1, 2, and 3 (Hercules, CA, USA) were used and analyzed at the start and end of each analytical run and again after every 10 samples. The lower limit of quantification (LOQ) for cadmium was 2.67 nmol/L. Values below the LOQ were assigned to the LOQ for analysis.

### 2.5. Statistical Analysis

To evaluate the factors associated with elevated BCL in the CKD population with low-level environmental exposure, we divided all the patients enrolled into three equal subclasses according to the tertiles of BCL values. The data from the three subclasses (i.e., low, middle, and high BCL) were compared and analyzed. Categorical variables were presented as numbers with percentages and analyzed using the chi-squared test. Depending on the normality of data distribution examined by the Kolmogorov-Smirnov method, continuous variables were presented as means with standard deviations or as medians with interquartile ranges (IQRs), and the independent t-test or Kruskal-Wallis H-test was used for univariate analysis. All variables with a *p* value ≤ 0.05 in univariate analyses were assessed by multinomial logistic regression analysis and multiple linear regression analysis to determine the factors independently associated with BCL, with age, diabetes, and other covariates adjusted by enter method. The effects of significant factors recognized in multivariate analyses were also examined in specific subgroups, such as non-dialysis CKD patients, hypertensive patients, men, and non-smokers. The statistical significance was set at *p* ≤ 0.05. Statistical Product and Service Solutions (SPSS) software (version 22.0; IBM, Armonk, NY, USA) was used for all analyses.

## 3. Results

### 3.1. Demographic and Clinical Characteristics of Enrolled Patients

We enrolled 200 adult patients in the study, including 41.00% with CKD stage 3a–b, 43.50% with CKD stage 4–5, and 15.50% with CKD stage 5D (i.e., HD patients). All the HD patients received intermittent HD three times a week according to the standard protocol in the same HD unit of the hospital. The demographic, environmental, lifestyle and biochemical profiles are provided in Table 1 and Table 2. The median age of the cohort was 67 years and women accounted for 35.50% of all patients. The most common comorbidities were hypertension (85.50%), followed by dyslipidemia (80.00%), degenerative or osteoporotic bone disease (75.50%), gout or hyperuricemia (48.00%), diabetes (24.50%), vascular disease (24.50%), heart failure (13.00%), and COPD (8.00%). There were 13.00% of patients with a history of malignancy and only a few patients with transplantation history (kidney transplant 3.00%; extrarenal transplant 1.00%). Statin use was noted in 45.00% of the patients. In addition, 42.50% of the patients received three or more types of antihypertensives, and 17.00% of patients took at least one type of glucose-lowering agent. The median eGFR was 28.80 mL/min/1.73 m^2^ (IQR, 16.20–42.00 mL/min/1.73 m^2^) and the median BCL was 6.36 nmol/L (IQR, 4.06–9.22 nmol/L). The cadmium levels in the RO water samples from the HD center were all <0.06 nmol/L between 2020 and 2021, far below the American Association for Advancement of Medical Instrumentation standard (<8.90 nmol/L) [26].

### 3.2. Differences of Demographic and Clinical Characteristics between Three BCL Subclasses

To analyze the factors associated with elevated BCL in the study population, we divided all the patients into three subclasses based on the tertiles of the BCL values, namely, the low BCL (≤4.77 nmol/L, *n* = 67), the middle BCL (4.78–7.90 nmol/L, *n* = 66), and the high BCL (≥7.91 nmol/L, *n* = 67) subclasses. The distribution of the demographic, environmental, lifestyle, and biochemical profiles in the three subclasses is provided in Table 1 and Table 2. The percentage of female sex was higher in the middle and high BCL subclasses (19.40%, 39.39%, and 47.76%, respectively, *p* = 0.002); additionally, smoking was more common in the high BCL subclass (7.46%, 6.06%, and 25.37%, respectively, *p* = 0.001). Compared to the other subclasses, the high BCL subclass had more late-stage CKD patients, especially CKD stage 5D (1.49%, 12.12% and 32.84%, respectively, *p* < 0.001), and less patients with CKD stage 3a–b (50.75%, 46.97%, and 25.37%, respectively, *p* = 0.006); furthermore, the percentage of CKD stage 5D was higher in the middle BCL subclass than in the low BCL subclass. Compared to the middle BCL subclass, the patients in the high BCL subclass had a higher burden of antihypertensive agents (≥3 types of antihypertensives, 38.81%, 31.82%, and 56.72%, respectively, *p* = 0.011). Additionally, statin use was more frequent in the low BCL subclass than in the high BCL subclass (59.70%, 43.94%, and 31.34%, respectively; *p* = 0.004). Other demographic, environmental, and lifestyle profiles, such as age, diabetes, BMI, other comorbidities, use of glucose-lowering agents, dietary preference, herb or cosmetics usage, traffic habit, occupational status, and living area were not significantly different between the subclasses. In the univariate analysis of biochemical data, the blood albumin level of the high BCL subclass was significantly lower than that of the low BCL subclass (median [IQR], 44.00 [41.38–46.23], 43.20 [40.90–45.00], and 42.05 [40.00–44.00] g/L, respectively, *p* = 0.021). In contrast, the blood phosphate level was significantly higher in the high BCL subclass compared to the low BCL subclass (median [IQR], 1.23 [1.07–1.36], 1.26 [1.10–1.49], and 1.29 [1.16–1.74] mmol/L, respectively, *p* = 0.034). Other biochemical data, such as HbA1c, UPCR, transferrin saturation, lipid profiles, SCr, eGFR, electrolytes, hemoglobin, uric acid, and liver enzymes were similar between three BCL subclasses.

### 3.3. Identification of BCL Associated Factors and Related Deviations of BCL

**Figure 1:**
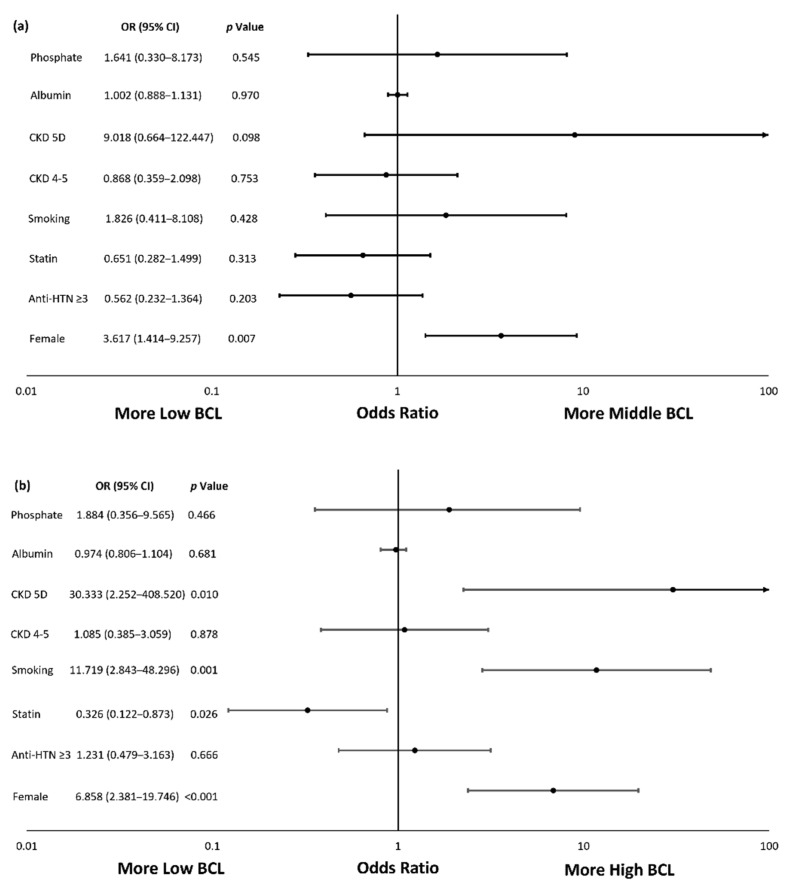
Forest Plots of BCL Associated Factors in CKD patients. (**a**) Middle BCL vs. Low BCL assessed by multinomial logistic regression analysis; (**b**) High BCL vs. Low BCL assessed by multinomial logistic regression analysis. Age, diabetes, and covariates with a *p* value ≤ 0.05 in univariate analyses were adjusted for analysis. Anti-HTN ≥ 3, antihypertensives ≥ 3 types; BCL, blood cadmium level; CKD, chronic kidney disease; CKD 5D, chronic kidney disease stage 5D; CKD 4–5, chronic kidney disease stage 4–5; CI, confidence interval; OR, odds ratio.

In the multinomial logistic regression analysis, after adjusting for age, diabetes, and covariates with a *p* value ≤ 0.05 in univariate analyses, female sex, smoking, and CKD stage 5D were the independent risk factors associated with high BCL (odds ratio [OR; 95% confidence interval, CI], 6.858 [2.381–19.746], *p* < 0.001, 11.719 [2.843–48.296], *p* = 0.001, and 30.333 [2.252–408.520], *p* = 0.010, respectively), and statin use was negatively correlated with high BCL (OR [95% CI], 0.326 [0.122–0.873], *p* = 0.026). Additionally, the female sex was also an independent risk factor for middle BCL (OR [95% CI], 3.617 [1.414–9.257], *p* = 0.007) (Table 3 and Figure 1). In the multiple linear regression analysis with age, diabetes, and covariates with a *p* value ≤ 0.05 in univariate analyses adjusted (Table 4), female sex, smoking, and CKD stage 5D were associated with elevations in BCL of 2.66 nmol/L (95% CI, 1.33–4.00, *p* < 0.001), 3.68 nmol/L (95% CI, 1.81–5.56, *p* < 0.001), and 3.38 nmol/L (95% CI, 0.95–5.82, *p* = 0.007), respectively. Statin was associated with a decline in BCL of 2.07 nmol/L (95% CI, −3.35–−0.78, *p* = 0.002).

### 3.4. The Effects of BCL Associated Factors in Specific Subgroups

**Figure 2:**
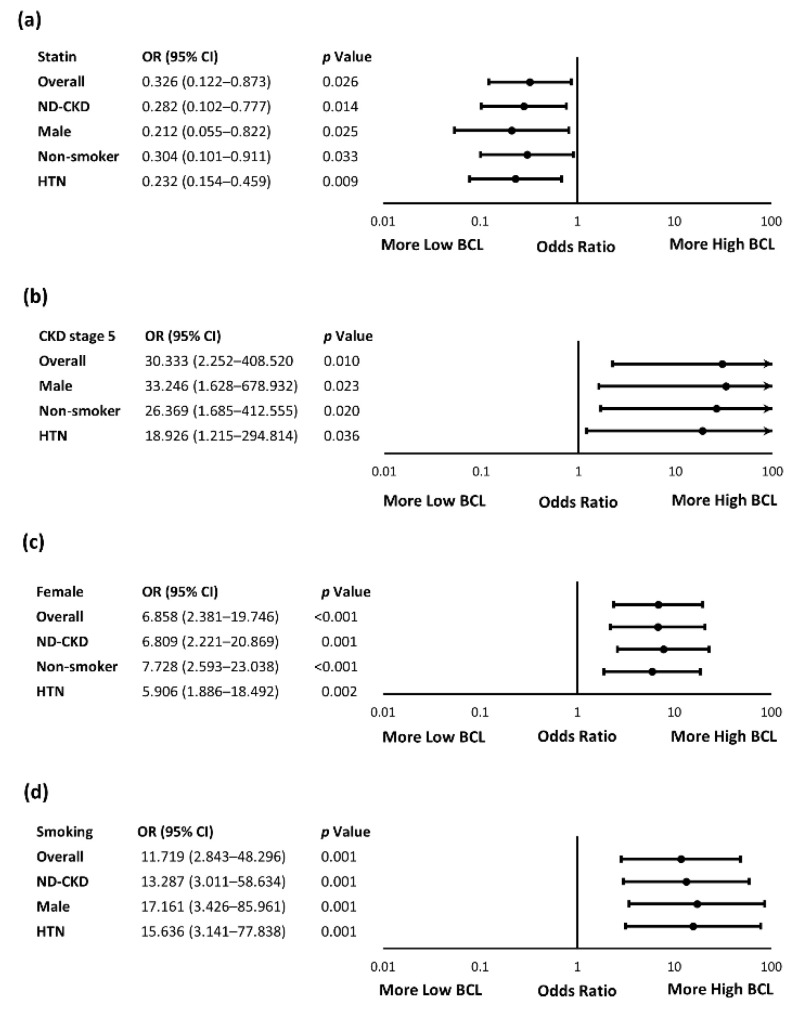
The Effects of BCL Associated Factors in Specific Subgroups. (**a**) Statin effects in subgroups; (**b**) CKD Stage 5D effects in subgroups; (**c**) female sex effects in subgroups; (**d**) smoking effects in subgroups. The numbers of specific subgroups were: ND-CKD (*n* = 179), male sex (*n* = 129), non-smokers (*n* = 174), and HTN (*n* = 171), respectively. HTN, hypertension; ND-CKD, non-dialysis CKD.

In the subgroup analyses (Figure 2), female sex, smoking, CKD stage 5D, and statin were still the factors significantly correlated with BCL in all the analyzed subgroups, including non-dialysis CKD, hypertensive patients, non-smokers, and men. The ORs of high BCL associated with smoking, CKD stage 5D, and statin increased slightly in male patients (OR [95% CI], 17.161 [3.426–85.961], *p* = 0.001, 33.246 [1.628–678.932], *p* = 0.023, and 0.212 [0.055–0.822], *p* = 0.025, respectively). On the other hand, the OR of high BCL associated with female sex increased slightly in non-smokers (OR [95% CI], 7.728 [2.593–23.038], *p* < 0.001).

## 4. Discussion

In our study, female sex, smoking, and late-stage CKD, especially CKD stage 5D, were identified as the independent risk factors for elevated BCL in the CKD population with low-level environmental cadmium exposure. In contrast, statin use was inversely correlated with high BCL in this population. In the subgroup analyses, these factors were still independently correlated with BCL in all the analyzed subgroups, including non-dialysis CKD, hypertensive patients, non-smokers, and men. The associations of BCL with other medical (including dialysis water contamination), demographic, environmental, and lifestyle factors in CKD patients were not proven in our study. There have been studies indicating that low-level environmental cadmium exposure has harmful effects on human health and complex interactions with other environmental contaminants; additionally, the blood cadmium elevation also deteriorates the risks and outcomes of CKD [8,20,22,27]. In our study, the median BCL of the total population was 6.36 nmol/L, which was higher than that in studies in the general Asian population (2.67–4.45 nmol/L) [12] and consistent with previous studies in dialysis-dependent patients [22,23], suggesting that CKD patients might be more vulnerable to low-level cadmium exposure in their daily lives and have a higher risk of cadmium accumulation.

In the general population, occupational exposure, living in industrial region, old age, smoking, dietary preference, female sex, and iron deficiency were identified as the risk factors for cadmium exposure or elevated blood cadmium in the literature [12,13,28,29]. However, specific risk factors for elevated blood cadmium in CKD patients have not been studied, and our research focused on the patients with CKD stage 3a–5D that are considered more vulnerable to cadmium exposure due to reduced renal excretion. In our analysis, the independent risk factors for elevated BCL in the CKD population included female sex, smoking, and late-stage CKD, especially CKD stage 5D. The sex differences in blood and urinary cadmium levels have been observed in epidemiologic studies, and female patients were prone to have higher body cadmium accumulation and higher risks of cadmium-associated toxicity in these studies [30,31]. A possible explanation for these sex-related differences is the higher frequency of iron deficiency in female patients, since iron and cadmium share the same intestinal transporter (i.e., divalent metal transporter 1 [DMT-1]) [32]. In addition to intestines, the renal proximal tubular cells also express DMT-1 exclusively in the membranes of late endosomes and lysosomes, and hence DMT-1 could play a role in the cadmium-associated nephrotoxicity as a route of cadmium efflux into tubular cell cytosol [33]. Owing to the upregulation of DMT-1, the accumulation and toxicity of cadmium might be enhanced with iron deficient conditions such as pregnancy and menstruation [34]. However, Kim et al. reported an analysis based on the Korean National Health and Nutritional Examination Survey and indicated that the blood cadmium was correlated with iron storage only in pre-menopausal females, although the blood cadmium was still higher in overall female patients [13]. Additionally, the BCL values of pregnant women were unexpectedly lower than those of nonpregnant women irrespective of iron storage in a study in China [35]. Furthermore, there had been little studies to assess the relationship between cadmium accumulation and iron status in CKD patients, and the expressions of DMT-1 were inconsistent in preclinical CKD models [34,36]. In our study, the female sex was positively correlated with BCL in CKD patients after adjusting for covariates and increased the risk of high BCL nearly 7 times, with a 2.66 nmol/L elevation of BCL. Because most female patients enrolled in our study were beyond the age of menopause and transferrin saturations were similar between the BCL subclasses, the sex difference in blood cadmium in CKD patients might be attributed to causes other than iron deficiency or menstrual blood loss. In addition, some gender-related practices such as consumption of cosmetics, herbal remedies and personal care products have been noticed as potential sources of toxic metal exposure in the literature [37]. In a recent report in Iran, associations between duration of using cosmetics and BCL had been recognized in young women, which supported the roles of gender-related factors in cadmium exposure [38]. In our analysis, the ratios of cosmetics usage and herbal consumption were similar between BCL subclasses, and the differences between the previous reports and our study might be partly related to the older age of our cohort and the limitations of questionnaire-based investigation about lifestyle factors. Though further investigations are warranted, our study highlighted the susceptibility of female CKD patients to cadmium accumulation even without iron deficiency, and the gender-related factors associated with cadmium accumulation in this population will be topics of interest in the future.

Tobacco smoking is a well known source of chronic toxic metal exposure including cadmium, and the BCL of smokers is 4–5 times higher than that of non-smokers [9,39]. The cadmium concentrations in tobacco fillers range from 0.29 to 0.54 μg/g and the cadmium contents in mainstream cigarette smoke range from 1.60 to 295 ng/cigarette in different analyses, making smoking behavior a major risk of cadmium toxicity in daily lives [40]. In our study, CKD patients with smoking behavior accounted for only 13.00% of the overall population, however, 25.37% of the high BCL subclass, and smoking was independently correlated with high BCL, which increased the risk of high BCL nearly 12 times with a 3.68 nmol/L increase in BCL. Since the associations between cadmium exposure and other cardiovascular risks have been found in the literature, such as dyslipidemia, inflammatory markers, and hypertension [41,42], the possible synergistic effects of cadmium exposure and smoking, another cardiovascular risk factor, on the outcomes of CKD are important issues for further research, and abstinence from smoking is essential to avoid cadmium toxicity.

In the human body, the kidney is the major organ of accumulation and the main elimination route of cadmium, and previous studies have suggested that BCL is positively associated with renal dysfunction [1,43,44]. Our study demonstrates that late-stage CKD was the major risk factor for elevated BCL in the CKD population even after adjustment for age and other covariates; additionally, the risk of high BCL was 30 times higher in CKD stage 5D compared with stage 3a–b; besides, CKD stage 5D was associated with an increase in BCL up to 3.38 nmol/L. The especially high risk of cadmium accumulation in CKD stage 5D could not be attributed to dialysis water contamination or lifestyle factors in our analysis, and therefore might be directly related to the severity of renal dysfunction. As the nephrotoxic potential of cadmium has been widely recognized, the progression of CKD could further amplify the deteriorative effects of cadmium on renal outcomes; hence, the vicious cycle requires particular attention and intervention [45]. On the other hand, though the inverse relationship between eGFR and body cadmium burden had been recognized in some studies, other studies indicated that SCr-based eGFR might be not associated or even paradoxically elevated with surrogates of body cadmium burden [46,47]. A possible explanation of the inconsistency is the time lag between cadmium-induced renal injury and absolute loss of viable nephrons. In our analysis, conventional renal biomarkers such as UPCR and eGFR were not significantly correlated with BCL, which also highlighted the roles of novel renal biomarkers such as N-acetyl-D-glucosaminidase (NAG), kidney injury molecule-1 (KIM-1) and 8-hydroxy-2-deoxyguanosine (8-OHdG) in the further research of cadmium-associated nephrotoxicity and tubulopathy [29,48].

In our study, the statin use was inversely correlated with high BCL even after adjusting for other covariates; moreover, the risk of high BCL was about one-third lower in statin users with a 2.07 nmol/L reduction of BCL, which has not been reported before. The association between statin use and BCL was independent of the lipid profile or underlying dyslipidemia. Statins, a group of β-hydroxy-β-methylglutaryl-CoA (HMG-CoA) reductase inhibitors, have pleiotropic roles in human diseases, including lipid-lowering effects, antioxidant properties, and anti-inflammatory activities [49]. Animal studies have indicated that statins might ameliorate cadmium-associated toxicity via anti-inflammatory and antioxidant mechanisms. In addition, statins have been found to interfere with the expression of the metallothionein 2A gene in a few in vitro studies [50,51]. Since metallothionein is a critical protein involved in the metabolism of several heavy metals, including cadmium, and the protective roles of metallothionein in cadmium toxicity have been recognized in animal studies [52], the links between statin use and the accumulation or toxicity of cadmium warrant further experimental and clinical investigations.

The subgroup analyses of our study demonstrates that sex differences with regard to BCL tended to increase in non-smokers; moreover, the influences of smoking, statin, and CKD stage 5D on BCL increased slightly in CKD patients who were men. A previous study also revealed that the differences in BCL between women and men in non-smokers were more prominent than in smokers (3.51 vs. 2.15 nmol/L); the elevation of BCL with smoking was more obvious in men than in women (4.57 vs. 3.22 nmol/L) [13]. Although dietary preference is a major determinant of cadmium exposure in non-smokers [53], the sex difference in BCL was not associated with the dietary pattern in our study. Therefore, further research is required to clarify the interactions between sex difference and smoking status on BCL in CKD patients. Despite the higher cadmium burden in female patients and smokers, a recent study indicated that the differences narrowed with renal function deterioration, which highlighted the importance of renal function in BCL [54]. Our analysis suggested that the impact of CKD severity on BCL might increase slightly in patients who are men; hence, the risk of cadmium accumulation in CKD patients who are men is worth noting. Our study also demonstrated a possible protective effect of statins on the BCL of patients with CKD, and the benefit seemed elevated in men. Although a recent review indicated that the beneficial effects of statins in cardiovascular disease might be attenuated in women [55], the paucity of research makes the interaction between statin and cadmium a notable issue; furthermore, the sex differences of statin effects on BCL also merit attention.

There were limitations to our study. Lifestyle and environmental characteristics, except smoking, were not identified as independent risk factors for high BCL in this CKD study. Diet is the major source of cadmium in low-level environmental exposure settings, and rice has been viewed as the leading source of cadmium burden in Asian populations [45,56]. In our study, only Asian patients were enrolled, and all the patients consumed rice as their staple food; therefore, the differences in dietary cadmium exposure might be minimized. Moreover, available tools for food assessment such as Food Frequency Questionnaire are of limited utility in dietary cadmium estimation, making the accurate evaluation of dietary factors (such as the individual amount or source of rice) more difficult [57]. Although occupational exposure, air pollution, contaminated water, living in industrial region, cosmetics, paint, and herbs have been identified as the potential sources of cadmium in the daily life [1,10,11,29,58], there were no differences in the relevant factors between BCL subclasses in our study, probably due to the exclusion of known occupational or accidental heavy metal poisoning at enrollment, the limitation of questionnaire-based investigation, and the advance of legislative regulations. Blood and urinary renal biomarkers were similar between BCL subclasses in our analysis, and hence longitudinal studies are required to clarify the impact of cadmium accumulation on renal biomarkers and outcomes, in which the novel renal biomarkers such as KIM-1, NAG and 8-OHdG could be utilized. In addition, CKD patients with eGFR > 60 mL/min/1.73 m^2^ were not included in our study, as well as PD patients, and specific research in the populations is crucial in the future. The zinc supplementation, which might be protective for cadmium toxicity [40], could not be precisely evaluated with our study design as the result of the large diversity of potential sources, including diet, healthy food, and over-the-counter medicines, and hence requires further research. Finally, due to the single-center and cross-sectional designs, the generalizability of our results could be limited by the relatively small sample size and potential confounding factors such as the medical practices of individual clinicians, unidentified gender-related factors, and unpredictable dietary influences. Despite these limitations, our study could improve the research gap about the risk factors of elevated BCL in the CKD population, especially those with late-stage CKD, and will be valuable for clinical CKD care. Large-scale prospective and longitudinal investigations are needed to verify our findings and elucidate the influence of cadmium on the CKD outcomes.

## 5. Conclusions

In conclusion, our study indicates that female sex, smoking, and late-stage CKD, especially CKD stage 5D, were the major risk factors for elevated blood cadmium in CKD patients, and statin use was negatively associated with blood cadmium in this population. These factors were also independently correlated with blood cadmium in subgroups, including non-dialysis CKD, hypertensive patients, non-smokers, and male patients. Further studies are required to verify these results.

## Figures and Tables

**Table 1:** Differences in Demographic Profiles and Clinical Characteristics Between Three BCL Subclasses.

	Total Patients	Low BCL	Middle BCL	High BCL	Univariate
		≤4.77 nmol/L	4.78–7.90 nmol/L	≥7.91 nmol/L	Analysis
	(*n* = 200)	(*n* = 67)	(*n* = 66)	(*n* = 67)	*p* Value
Age (year), median (IQR)	67 (58–76)	66 (58–76)	69 (61–74)	66 (57–76)	0.659
Female, *n* (%)	71 (35.50)	13 (19.40)	26 (39.39) ^#^	32 (47.76) ^#^	0.002 *
BMI (kg/m^2^), median (IQR)	24.73 (22.15–27.68)	24.46 (21.63–28.08)	24.96 (22.91–28.17)	24.74 (22.15–26.67)	0.276
Hypertension, *n* (%)	171 (85.50)	52 (77.61)	60 (90.91)	59 (88.06)	0.072
Diabetes Mellitus, *n* (%)	49 (24.50)	12 (17.91)	23 (34.85)	14 (20.90)	0.059
Dyslipidemia, *n* (%)	160 (80.00)	56 (83.58)	54 (81.82)	50 (74.63)	0.390
Vascular Disease, *n* (%)	49 (24.50)	13 (19.40)	16 (24.24)	20 (29.85)	0.372
Heart Failure, *n* (%)	26 (13.00)	6 (8.96)	9 (13.64)	11 (16.42)	0.431
COPD, *n* (%)	16 (8.00)	3 (4.48)	5 (7.58)	8 (11.94)	0.278
Gout or Hyperuricemia, n (%)	96 (48.00)	36 (53.73)	33 (50.00)	27 (40.30)	0.275
Bone Disease ^@^, *n* (%)	151 (75.50)	47 (70.15)	49 (74.24)	55 (82.09)	0.264
Kidney Transplant, *n* (%)	6 (3.00)	1 (1.49)	3 (4.55)	2 (2.99)	0.587
Extrarenal Transplant, *n* (%)	2 (1.00)	1 (1.49)	1 (1.52)	0 (0.00)	0.601
Previous Malignancy, *n* (%)	26 (13.00)	9 (13.43)	8 (12.12)	9 (13.43)	0.967
CKD Stage 3a–b, *n* (%)	82 (41.00)	34 (50.75)	31 (46.97)	17 (25.37) ^#,$^	0.006 *
CKD Stage 4–5, *n* (%)	87 (43.50)	32 (47.76)	27 (40.91)	28 (41.79)	0.705
CKD Stage 5D, *n* (%)	31 (15.50)	1 (1.49)	8 (12.12) ^#^	22 (32.84) ^#,$^	<0.001 *
Anti-HTN ≥3 types, *n* (%)	85 (42.50)	26 (38.81)	21 (31.82)	38 (56.72) ^$^	0.011 *
Glucose-lowering Agent, *n* (%)	34 (17.00)	10 (14.93)	16 (24.24)	8 (11.94)	0.144
Statin Usage, *n* (%)	90 (45.00)	40 (59.70)	29 (43.94)	21 (31.34) ^#^	0.004 *

* *p* ≤ 0.05; ^#^ significantly different compared to the low BCL subclass; ^$^ significantly different compared to the middle BCL subclass; ^@^ degenerative/osteoporotic bone disease, such as osteoporosis/osteopenia/osteoarthritis. Anti-HTN, antihypertensives; BCL, blood cadmium level; BMI, body mass index; CKD, chronic kidney disease; COPD, chronic obstructive pulmonary disease; IQR, interquartile range; *n*, number.

**Table 2:** Differences in Environmental, Lifestyle and Biochemical Profiles Between Three BCL Subclasses.

	Total Patients	Low BCL	Middle BCL	High BCL	Univariate
		≤4.77 nmol/L	4.78–7.90 nmol/L	≥7.91 nmol/L	Analysis
	(*n* = 200)	(*n* = 67)	(*n* = 66)	(*n* = 67)	*p* Value
Environmental and Lifestyle Profiles, *n* (%)					
Drinking	24 (12.00)	9 (13.43)	7 (10.60)	8 (11.94)	0.881
Smoking	26 (13.00)	5 (7.46)	4 (6.06)	17 (25.37) ^#,$^	0.001 *
Betelnut Usage	16 (8.00)	6 (8.96)	2 (3.03)	8 (11.94)	0.142
Seafood Preferred ^a^	72 (36.00)	25 (37.31)	22 (33.33)	25 (37.31)	0.814
Larger Fish Preferred ^a^	50 (25.00)	19 (28.36)	15 (22.73)	16 (23.88)	0.749
Organ Meat Preferred ^b^	19 (9.50)	7 (10.45)	7 (10.61)	5 (7.46)	0.817
Traffic by Walk/Scooter	142 (71.00)	50 (74.63)	47 (71.21)	45 (67.16)	0.784
Urban Area	153 (76.50)	52 (77.61)	48 (72.73)	53 (79.10)	0.663
Petrochemical Region	156 (78.00)	54 (80.60)	52 (78.79)	50 (74.63)	0.694
Occupation of Risk ^c^	38 (19.00)	15 (22.39)	14 (21.21)	9 (13.43)	0.404
Herbal Usage	41 (20.50)	13 (19.40)	13 (19.70)	15 (22.39)	0.847
House Age >10 years	184 (92.00)	62 (92.54)	61 (92.42)	61 (91.04)	0.940
Well/Spring Water Usage	63 (31.50)	20 (29.85)	25 (37.88)	18 (26.87)	0.417
Cosmetics Usage	70 (35.00)	21 (31.34)	21 (31.81)	28 (41.79)	0.282
Biochemical Profiles, median (IQR)					
SCr (μmol/L)	152.50 (114.38–251.63)	160.13 (114.38–282.13)	137.25 (106.75–228.75)	152.50 (122.00–236.38)	0.492
eGFR (mL/min/1.73 m^2^)	28.80 (16.20–42.00)	30.60 (15.00–40.80)	33.00 (16.80–43.20)	27.00 (18.00–39.00)	0.600
UPCR (mg/mmol)	58.76 (15.30–126.97)	58.44 (17.56–163.88)	48.42 (9.72–121.99)	68.30 (19.24–137.07)	0.657
Hemoglobin (g/L)	113.0 (104.0–130.0)	120.50 (104.00–133.00)	112.00 (103.50–129.25)	112.00 (104.00–128.25)	0.335
Glycated Hemoglobin (%)	5.90 (5.50–6.30)	5.60 (5.44–6.20)	5.90 (5.70–6.50)	5.90 (5.50–6.18)	0.151
TC (mmol/L)	4.27 (3.73–4.92)	4.35 (3.76–4.85)	4.16 (3.61–4.90)	4.27 (3.68–5.23)	0.508
LDL-C (mmol/L)	2.41 (1.89–2.91)	2.42 (1.87–2.81)	2.32 (1.89–2.89)	2.44 (1.89–3.37)	0.402
HDL-C (mmol/L)	1.19 (0.98–1.50)	1.20 (0.98–1.55)	1.17 (0.98–1.50)	1.24 (0.98–1.48)	0.897
Triglyceride (mmol/L)	1.20 (0.80–1.66)	1.23 (0.80–1.67)	1.13 (0.80–1.68)	1.27 (0.91–1.60)	0.782
ALT (ukat/L)	0.28 (0.20–0.40)	0.28 (0.18–0.38)	0.28 (0.20–0.38)	0.27 (0.20–0.42)	0.928
Albumin (g/L)	43.00 (40.80–45.25)	44.0 (41.38–46.23)	43.20 (40.90–45.00)	42.05 (40.00–44.00)^#^	0.021 *
Uric Acid (mmol/L)	0.38 (0.32–0.44)	0.37 (0.33–0.43)	0.38 (0.29–0.43)	0.39 (0.33–0.46)	0.329
Ca (mmol/L)	2.33 (2.25–2.40)	2.30 (2.23–2.38)	2.35 (2.25–2.43)	2.33 (2.25–2.38)	0.440
P (mmol/L)	1.26 (1.10–1.49)	1.23 (1.07–1.36)	1.26 (1.10–1.49)	1.29 (1.16–1.74) ^#^	0.034 *
K (mmol/L)	4.50 (4.10–4.90)	4.50 (4.10–4.80)	4.55 (4.28–4.90)	4.35 (4.10–4.93)	0.285
Transferrin Saturation (%)	32.46 (25.77–40.62)	33.76 (25.00–41.16)	30.90 (26.82–39.71)	32.52 (25.38–43.54)	0.829

^a^ defined as intake ≥4 times/week and large fish defined as tuna, shark, swordfish, or related products; ^b^ defined as intake ≥3 times/day; ^c^ defined as current or previous work about manufacturing, fuel, building, and other occupations with higher possibility of metal contact; * *p* ≤ 0.05; ^#^ significantly different compared to low BCL subclass; ^$^ significantly different compared to middle BCL subclass. ALT, alanine aminotransferase; Ca, blood calcium; eGFR, estimated glomerular filtration rate; HDL-C, high-density lipoprotein cholesterol; K, blood potassium; LDL-C, low-density lipoprotein cholesterol; P, blood phosphate; SCr, serum creatinine; TC, total cholesterol; UPCR, urinary protein/creatinine ratio.

**Table 3:** Multinomial Logistic Regression Analysis of BCL Associated Factors.

	Middle BCL vs. Low BCL		High BCL vs. Low BCL	
	Adjusted OR ^#^ (95% CI)	*p* Value	Adjusted OR ^#^ (95% CI)	*p* Value
Female	3.617 (1.414–9.257)	0.007 *	6.858 (2.381–19.746)	<0.001 *
Anti-HTN ≥3 types	0.562 (0.232–1.364)	0.203	1.231 (0.479–3.163)	0.666
Statin Usage	0.651 (0.282–1.499)	0.313	0.326 (0.122–0.873)	0.026 *
Smoking	1.826 (0.411–8.108)	0.428	11.719 (2.843–48.296)	0.001 *
CKD Stage 4–5	0.868 (0.359–2.098)	0.753	1.085 (0.385–3.059)	0.878
CKD Stage 5D	9.018 (0.664–122.447)	0.098	30.333 (2.252–408.520)	0.010 *
Albumin (g/L)	1.002 (0.888–1.131)	0.970	0.974 (0.860–1.104)	0.681
Phosphate (mmol/L)	1.641 (0.330–8.173)	0.545	1.844 (0.356–9.565)	0.466

* *p* ≤ 0.05; ^#^ age, diabetes, and covariates with a *p* value ≤ 0.05 in univariate analyses were adjusted for analysis. CI, confidence interval; OR, odds ratio.

**Table 4:** Multiple Linear Regression Analysis of BCL (nmol/L) Associated Factors.

	β	SE	Standardized β	95% CI for β	*p* Value
Female	2.66	0.68	0.27	1.33–4.00	<0.001 *
Anti-HTN ≥3 types	0.33	0.64	0.03	−0.94–1.59	0.611
Statin Usage	−2.07	0.65	−0.22	−3.35–−0.78	0.002 *
Smoking	3.68	0.95	0.27	1.81–5.56	<0.001 *
CKD Stage 4–5	−0.12	0.71	−0.01	−1.52–1.29	0.870
CKD Stage 5D	3.38	1.23	0.28	0.95–5.82	0.007 *
Albumin (g/L)	−0.10	0.09	−0.08	−0.28–0.08	0.264
Phosphate (mmol/L)	0.36	1.03	0.03	−1.67–2.38	0.728

Age, diabetes, and covariates with a *p* value ≤ 0.05 in univariate analyses were adjusted for analysis. * *p* ≤ 0.05. β, regression coefficient; SE, standard error.

## Data Availability

All the data generated in this study are available from the corresponding author (leewc@cgmh.org.tw) upon reasonable request due to research regulation of the hospital.

## Supplementary Materials

The following are available online at https://www.mdpi.com/article/10.3390/ijerph182312337/s1, Table S1. Questionnaire for Environmental and Lifestyle Profiles.
