# Supplementary material for: The Risk Factors of Blood Cadmium Elevation in Chronic Kidney Disease
Source: Int J Environ Res Public Health. 2021 Nov 24;18(23):12337. doi: 10.3390/ijerph182312337 (PMC8656955; doi:10.3390/ijerph182312337)
Supplement: Supplementary file 1 [file ijerph-18-12337-s001.zip › ijerph-1458101-supplementary.pdf]

Table S1. Questionnaire for Environmental and Lifestyle Profiles

Personal Information

1.Sex: □Male □Female

2.Age: _____ year-old

3.Weight: _____kg

4.Height: _____cm

5.Address of residence: ______________________________

6.Occupation (current or previous if retired): ______________________________

Alcohol Consumption

1.Do you intake alcohol regularly ? □Yes □Abstinence □Never

2.If your answer is "Yes", the average amount of consumption: _____ drinks (=150ml beer)/week

3.If your answer is "Abstinence", the time of abstinence: □≤ 10 years □>10 years

4.If your answer is "Abstinence", previous average amount of consumption: _____ drinks/week

Smoking Habit

1.Do you smoke regularly ? □Yes □Abstinence □Never

2.If your answer is "Yes", the average amount of consumption: _____ packs (=20 cigarettes)/day

3.If your answer is "Abstinence", the time of abstinence: □≤ 10 years □>10 years

4.If your answer is "Abstinence", previous average amount of consumption: _____ packs/day

Betelnut Consumption

1.Do you intake betelnut regularly ? □Yes □Abstinence □Never

2.If your answer is "Yes", the average amount of consumption: _____ pieces/day

3.If your answer is "Abstinence", the time of abstinence: □≤ 10 years □>10 years

4.If your answer is "Abstinence", previous average amount of consumption: _____ pieces/day

Specific Food Preference

1.Your frequency of seafood (or related products) intake: □<2 □2-3 □4-6 □≥7 times/week

2.Your frequency of larger fish (tuna, shark, swordfish, or related products) intake : □<2 □2-3 □4-6 □≥7 times/week

3.Your frequency of organ meat (animal liver, stomach, colon, etc.) intake: □<1 □1-2 □≥3 times/day

Information of Daily Life

1.Your main daily traffic tool:

□Walk/bicycle □Scooter/motorcycle □Car/Van □Public transit (bus, MRT, light rail, etc.)

2.The age of your current residence (or the one you lived the longest within 10 years): □<5 □5-10 □>10 years

3.The source of your drinking water: □Tap water □Bottled water □Well water □Mountain spring water

4. Do you intake herbal remedy ? □Currently or regularly for at least 3 months within 10 years □ Never or seldom

5. Do you use cosmetics ? □Currently or regularly for at least 3 months within 10 years □ Never or seldom
